# Micronuclei and Other Nuclear Abnormalities in *Patella* Limpet as Biomarkers of Cytogenotoxicity in Recreational Port Waters

**DOI:** 10.1002/tox.70055

**Published:** 2026-02-23

**Authors:** Carlos Valiente‐Diaz, Eva Garcia‐Vazquez, Verónica Soto‐López

**Affiliations:** ^1^ Department of Functional Biology University of Oviedo Oviedo Spain; ^2^ Department of Nautical Science and Technology University of Oviedo Gijón Spain

**Keywords:** diesel, heavy metals, limpets, micronucleus, northern Spain, petrol

## Abstract

Over the past decades, the growth in human population and economic activities has led to an increase in maritime traffic. This rise puts additional pressure on marine environments, vessel fuel spills being considered to have a major impact on the ecosystem. In this study we conducted the micronucleus (MN) test on 48 limpets of the genus *Patella* between June and July 2024, from four marinas of the Asturias coast (SW Bay of Biscay) with different traffic volumes, water metal contents (As, Cd, Hg and Pb), and use of fuel types as determined from a survey to ship owners. We also conducted an experiment exposing limpets to recreational boat contaminants (petrol, diesel, and ecological soap) for 5 days, then analyzing MN and other nuclear abnormalities (NAs). Results revealed that limpets exposed to petrol exhibited the highest MN count, and the highest proportion of MN (over all the NA), followed by those exposed to diesel. The group treated with ecological soap did not differ significantly from the untreated control. Limpets from the biggest Gijón marina exhibited the highest MN count and the highest As concentration. In regression analysis, the level of MN and other NAs in limpets sampled from the marinas was explained directly by the city size, and by the sum of stressors including the proportion of boats using petrol. The proportion of MN over total abnormalities was correlated with the number of moorings. These results support the contribution of recreational boating to cytogenotoxicity in local communities of marine fauna, likely from fuel spills.

## 1 Introduction

In the last decades, human population and economic growth have led to the creation of new marine trade routes [1]. New industrial and urban structures have transformed most of the world's oceanic regions into maritime traffic hotspots [2]. It is estimated that approximately 120 000 vessels are used for trading between more than 5000 ports worldwide [3, 4]. Moreover, the recreational use of boats has also been reported to be a relevant factor in marine traffic at a local level [4, 5]. The total number of small craft, defined as vessels with a length of less than 24 m and a gross tonnage of up to 50 tons, stands at 28 million units worldwide encompassing a diverse range of vessels, including nautical boats, pleasure crafts, sailing boats, and small fishing boats [6].

This rise in maritime traffic puts more pressure on marine environments [7]. Of concern are discharges of wastewater [8]; air emissions [9, 10]; physical impacts such as noise [8] or animal collisions [11]; and fuel spills [12]. Among these, fuel spills are considered to have a major impact on marine ecosystems [13], as they can result in a big input of petrol to the aquatic environment. Petrol (gasoline) is mainly formed by noncyclic and cyclic hydrocarbons, sulfur compounds, alkylphenols, and heavy metals [14]. Since 1922, lead (Pb) has been used as an additive to improve engine performance in most of the petrol fuels [15]. Diesel is one of the main derivatives from crude oil distillation [16]. It is formed by aliphatic hydrocarbons (~64%), benzene and polycyclic aromatic hydrocarbons (PAHs) (~35%), and olefinic hydrocarbons (~1%–2%) [17]. Diesel fuel is frequently considered a more environmentally friendly alternative to petrol due to its higher energy efficiency and lower carbon dioxide emissions per unit of energy [18]. However, it remains a significant source of air pollutants, including nitrogen oxides and particulate matter, which contribute to environmental degradation and pose health risks [19]. Petrol and diesel differ in the content of heavy metals considered toxic such as arsenic (As), cadmium (Cd), mercury (Hg), and Pb; in Europe, the average concentration of these metals is much higher in petrol than in diesel [20]. Heavy metals are of special concern due to their persistence in the environments [21] and their genotoxic effects [22]. Cd, Pb, and Hg are considered the most toxic. Hg and Pb have strong genotoxic activity, depending on the compound's speciation in the case of Hg [23, 24]. International regulations as the Directive 2008/105/EC of the European Parliament and of the Council of 16 December 2008 establish maximum allowable concentrations of Cd, Hg, and Pb in water (EUR‐Lex).

Besides heavy metals, other products employed by small craft may also be genotoxic. For example, all the ship hulls must be periodically cleaned, but there is not much information about the genotoxicity of the products employed; although the genotoxic activity of general soap is not very high [25], this is an issue worth exploring.

Biomonitoring projects evidence a correlation between genotoxicity and chronic harmful effects to animal populations [26, 27]. Major increases in genotoxicity are observed in marine organisms from marine areas affected by fuel spills, and the effects last for years after the accidents [28]. According to the OSPAR Commission [29], genotoxicity is defined as “Potentially harmful effects on genetic material not necessarily associated with mutagenicity, may be indicated by induced damage to DNA without direct evidence of mutation.” Out of all the possible alternatives, the micronucleus (MN) test is one of the most used in vivo assays to measure genotoxicity [30], mainly due to its easiness [31, 32]. MN are small DNA fragments separated from the main nucleus that can be due to chromosome breakages (clastogenic) or irregular sorting of chromosomes during the mitosis (aneugenic) [30, 33]. The most known causes of MN are: factors responsible for chromosome lagging, including aberrant kinetochore‐microtubule attachments and defects in the mitotic spindle assembly [34], acentric chromosome fragments produced by DNA breaks which are not repaired [35], and a failure in the assembly of the nucleus and its envelope at the end of the mitosis [36]. MNs are usually found in diverse pathologies, including cancer [37], and can appear throughout natural processes such as aging [30]. They can also be induced by environmental factors and genotoxic compounds [38]. The MN test is useful to assess genotoxicity caused by heavy metals [39, 40], being a widely used indicator of DNA damage and genomic alterations [41], both in vitro genotoxicity testing [38] and in vivo biomonitoring studies [42]. It is gaining special relevance in biomonitoring studies measuring genotoxicity in aquatic environments [43]. Besides MN, other alterations of cell nuclei indicate genotoxicity, including blebbed, lobed and notched nuclear membranes [44], as shown in many studies [45, 46]. Blebbed and lobed nuclei are related to failures in the tubulin polymerization process [47], while notched nucleus is more related to aneugenic toxic activity [48].

Mollusks, especially bivalves, are considered good bioindicators for monitoring pollutants in coastal environments because of their resistance, sessile lifestyle when adults, easy sampling, and high accumulation of chemicals [31]. Mussels are most employed due to their filter‐feeding strategy and worldwide distribution [49, 50]. However, over the past years, other marine bioindicators have been proposed in environments where mussels are scarce or difficult to obtain [51]. Among them, gastropods such as limpets are being increasingly used for this purpose [52, 53, 54, 55]. Limpets of the genus *Patella* (Gastropoda, Patellidae) are marine herbivores that inhabit the intertidal zones from North Europe to the South of Angola [56]. They are considered key species in rocky intertidal communities because their grazing helps in maintaining the microalgae abundance [57] and controls the establishment of other organisms [58]. *Patella* limpets have been successfully employed as heavy metal bioindicators in the Canary Islands [51, 52], Northwest Spain [59] and Portugal [60]. A few studies have used the MN test in limpets to measure the genotoxic effects of different compounds [61, 62].

In this study we will use the MN test in *Patella* limpets to assess the environmental genotoxicity of waters from South Bay of Biscay marinas with different maritime traffic and usage of fuel and cleanser types. The relative genotoxicity of those products will be examined from experimental exposure in controlled conditions, for a better understanding of the results obtained in the wild. The departure hypothesis was that limpets from marinas with more maritime traffic are expected to exhibit more MN and nuclear abnormality counts. The main objectives of this study were

1. To investigate the use of fuel and cleanser types in marinas with different maritime traffic from Asturias (Southwest Bay of Biscay, Spain).
2. To evaluate the level of environmental genotoxicity in those marinas using *Patella* species as bioindicator and MN and other nuclear abnormalities as markers.
3. To experimentally compare the genotoxic effects of the most used fuels and cleaning products in the marinas analyzed.

## 2 Materials and Methods

### 2.1 Sampling Locations

Four marinas were included in this study (see map in Figure 1). Site 1 is the marina of Gijón, with a longstanding tradition of nautical recreation [63] and is located at two kilometers from Gijón's international port by sea. The international port of Gijón occupies a total area of 415 ha, with 7000 linear meters of quay, making it the biggest in the Asturias region [64]. By the middle of the year 2024, a total of 907 557 tons of cargo had already been handled at its facilities [65]. Additionally, the bay of Gijón is home to two marinas: the Marina Yachts and the recreational port of Gijón. The Marina Yachts has a 9000 m^2^ space dedicated to boat maintenance and a total of 160 moorings. The recreational port of Gijón, with its longstanding tradition of nautical recreation, has a total of 675 moorings.

**FIGURE 1:**
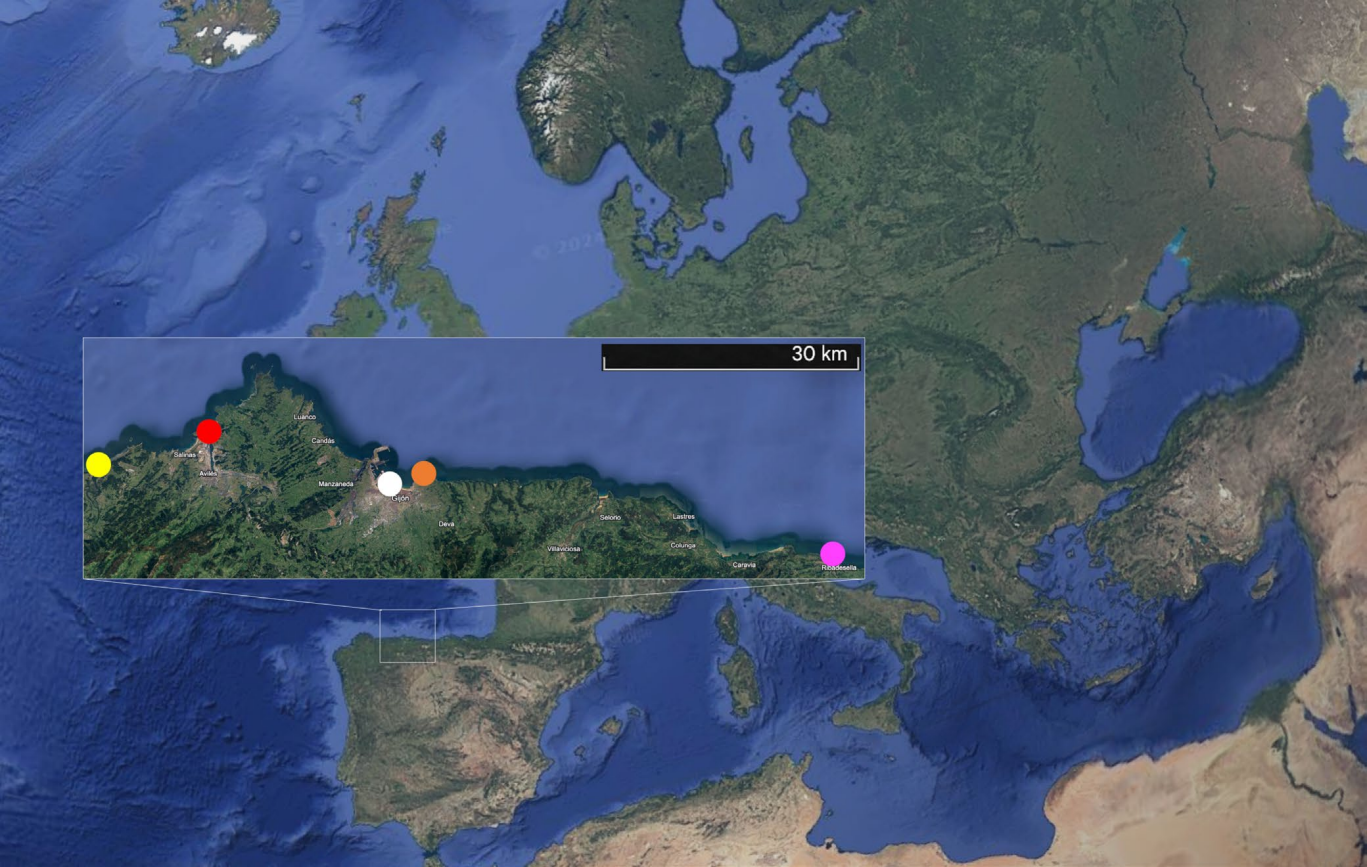
Map showing the five sampling sites. White: Gijón port (Site 1); red, Avilés port (Site 2); yellow, Nalón ports (Site 3); pink, Ribadesella port (Site 4); orange, El Rinconín beach (Site 5). Satellite images are extracted from Google Earth.

Site 2 is Avilés marina, located adjacent to the fishing port and in front of the commercial port of Avilés. The port's infrastructure enables it to handle approximately 6 million tonnes of cargo annually. As of July 2024, the port has handled a total of 262 831 tons of cargo [65]. The port is in an estuary and engages in annual dredging to maintain the marine draft [66]. The fishing port is the first in the region in terms of landings [67]. The marina with recreational boats, situated on the left bank of the estuary, is distributed across 16 piers for small leisure boats and offers approximately 200 berths [68].

Site 3 is situated in the Nalón estuary, where there are two fishing ports: San Juan de la Arena and San Esteban de Pravia, located on the east and west banks, respectively. In the past, the dock of San Esteban de Pravia's port used to be a primary port for transporting solid bulk materials, such as coal from the surrounding mining regions [69]. Nowadays, the area features a marina with berthing facilities for leisure for approximately 80 recreational boats and a separate berthing area for fishing vessels [70]. On the opposite bank of the river lies the fishing port of San Juan de la Arena. The port has a mooring area for approximately 100 moorings, distributed in one dock for small sport boats [71]. In the estuary, there is a third town named Soto del Barco with a small mooring area for 27 fishing boats.

Site 4 is placed near the marina of Ribadesella [72]. It is situated at the mouth of the Sella estuary. In the late 18th century, the port was used as a trading hub for various materials, such as wood and minerals [73]. Today, its primary use is recreational, with around 230 moorings.

The limpets employed in the experiment were sampled from Site 5 (in orange in the map of Figure 1), El Rinconín beach. It is located on the eastern side of Gijón's bay. The beach substrate is dark sand, rocks, and gravel. It is regarded as a cleaner point (at least in comparison with the marinas) because the beach is regularly cleaned up, and there are no industrial or urban sewages nearby [74].

### 2.2 Sample Collection

At sites 1–4, 12 limpets (*Patella cf. vulgata*) of similar size were collected from each site, following the recommendations of [75] that stated that at least 10 individuals are needed per sample site to reach an adequate statistical power to detect differences in MN frequency. For the experiment of exposure to products employed in the marinas, 48 limpets were obtained from Site 5. Limpets were gently detached from the rocks or port walls with a spatula and transported alive to the laboratory, in a closed box filled with marine water from the same site where they were located. At the same time, four samples of 5 mL of water were taken from different zones of each study site and stored frozen in the laboratory for further heavy metal quantification. For Nalón marinas, two samples of water were collected from San Esteban de Pravia, and two were collected from San Juan de la Arena marina area.

### 2.3 Heavy Metal Analysis of Water Samples

Quantification of four heavy metals (As, Cd, Hg, Pb, specifically, isotope ^208^Pb) was performed in the Scientific‐Technical Services of the University of Oviedo (Spain) as described in [76]. Briefly, water samples were directly loaded in an Agilent 7900 Series ICP‐MS. Samples were measured three times, and means and relative standard deviation (RSD) were calculated.

The limits of detection for these measurements were 0.0053 μg/kg for As, 0.0007 μg/kg for Cd, 0.0259 μg/kg for Hg, and 0.0006 μg/kg for Pb.

### 2.4 Surveys to Ship Owners

A total of 133 surveys were performed on the marinas of the study to ship owners docked there. They were asked to kindly answer four questions: boat type (three options: sailboat, < 8 m recreational boat, > 8 m recreational boat), fuel type (two options: petrol or diesel), boat hull cleaning products employed (free response), and who cleaned the boat (two options: owner or shipyard staff). The answers obtained are presented in Table S1.

### 2.5 Experimental Exposure to Products Employed by Ship Owners

The limpets were assigned to one of four groups (12 limpets per group). They were put in separate tanks filled with 20 L of marine water from the same location as the original limpets (Site 5, see Figure 1). The tank bottom was covered with rocks with a diameter between 8 and 14 cm, with encrusted green algae taken from the same site to ensure normal feeding. Limpets were left for 7 days with daily temperature monitoring (between 19.5°C and 21°C) and constant water aeration to acclimatize to laboratory conditions.

Based on the answers received from the surveys, three contaminants were assayed: petrol (95 RON), diesel (51 CN), and the most employed cleanser that was ecological soap (Aquatic mixture of oxalic acid, ethylene glycol, isopropyl alcohol, alcohol ethoxylates C11‐15 secondary, and hydrofluoric acid). According to the information provided by the manufacturer, the petrol here employed contained 5 mg/L of Pb, while no information about heavy metals content appeared in diesel.

A control group was established, where limpets were maintained in the same water in which they had acclimatized. Each contaminant was diluted and added separately in each experimental tank to a final concentration of 1 g/L. Fuel concentrations in surface water are usually lower than the proposed levels in this study [77, 78]. However, in cases of oil spills or areas with dense maritime traffic, concentration values tend to be higher. As an example, 3 years after an oil spill in the Malaysian region, concentration levels of petrol in the surface water range between 93 and 503 mg/L [79]. Therefore, a value of 1 g/L was chosen as a higher‐than‐usual exposure, yet still a plausible scenario, to ensure a clear experimental response. The limpets were left in the tanks for 5 days, during which time temperature was monitored daily, and water aeration was maintained consistently. They were carefully observed throughout the exposure period, and any limpet that died was promptly removed from the tank. At the end of the exposure experiment, all limpets were euthanized for gill extraction.

### 2.6 Slide Preparation and Nuclear Abnormality Count

Nuclear abnormalities were analyzed on gill cells. A portion of the gills was excised with a scalpel. As the limpets' gill tissue is quite tough, the excised gills were nipped with tweezers for 2 min. This process ensured the effective disruption of the tissue structure. Then, it was smeared along a slide forming a single layer of cells and left to air‐dry overnight. Two slides per sample were prepared and labeled with a code to identify the experimental tank.

The slides were initially fixed with absolute methanol for 3 min, then stained with 1:9 Giemsa diluted in phosphate‐buffered saline solution (PBS; pH = 7.2) for 10 min, left to dry, and mounted with DPX.

Stained slides were examined at a final magnification of ×1000 under an EcoBlue Blueline series microscope. From each slide, 1000 cells were scored. All slides were examined by a single observer in blind, that is, without looking at the slide code. Following [44], nuclear abnormalities were classified into five categories: MN; blebbed, lobed, notched nucleus; and other abnormalities (Figure 2). Micronuclei are round or ovoid non‐refractory particles of about one‐fifth the size of the primary nucleus that share the same coloration as the main cell nucleus (Figure 2A). Blebbed nuclei feature a minor evagination of the nuclear membrane, with high contents of euchromatin. Lobed nuclei are characterized by more pronounced evaginations compared to blebbed nuclei. Notched nucleus displays a significant cleft, lacking any nuclear material within it. For the purposes of this study, only those nuclei with cytoplasm and an apparently intact cell wall were considered.

**FIGURE 2:**
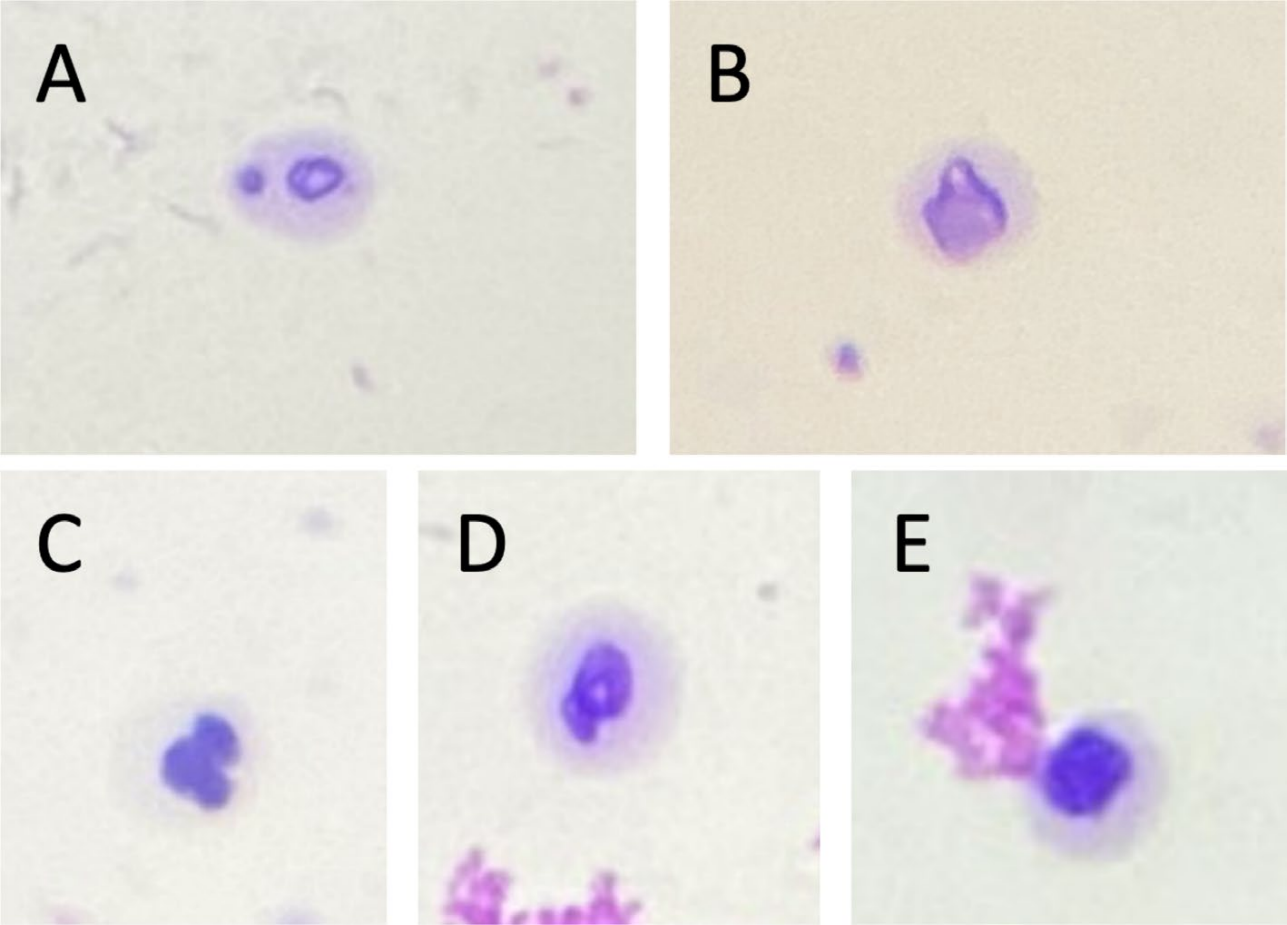
Photograph showing nuclear abnormalities found from *Patella vulgata* gill cells in this study. (A) Micronucleus; (B) blebbed nucleus; (C) lobbed nucleus; (D) notched nucleus; and (E) normal nucleus.

### 2.7 Statistical Analysis

The following variables were considered: nuclear abnormalities; heavy metal concentration in water; city population size (indicator of environmental disturbance, cytogenotoxicity in running waters is associated with city size in different regions, e.g., [80, 81]); number of marina moorings (indicator of maritime traffic); proportion of vessels using petrol (expectedly containing more heavy metals than other products). A composite variable called “Disturbance score” was constructed summing the rank of the three latter environmental variables for each site (4 corresponding to the maximum and 1 to the minimum values). Frequencies of MN and other nuclear abnormalities were expressed per 1000 cells.

Normality was checked with Shapiro–Wilk test, and homoscedasticity with Levene's test. ANOVA and post hoc Tukey's tests were employed to compare groups of samples, that is, among marinas or among experimental treatments. When the dataset did not fit ANOVA requirements, nonparametric tests were used for comparisons between groups: Kruskal–Wallis test, followed by pairwise Mann–Whitney post hoc test in case of significant differences.

Differences in heavy metals content in water samples from the four marinas were checked with ANOSIM analysis, using Euclidean distances and 9999 permutation numbers.

Comparisons between marinas for the proportion of boat owners' using a product, or between the proportion of the type of abnormalities among groups, were done using contingency chi‐square analysis, with Cramer's *V* as a proxy for effect size.

Association between biological variables (nuclear abnormalities) as dependent and environmental stressors as independent variables was tested using ordinary least square regressions (LSRs). For the limited number of marinas, the nonparametric Kendall's *τ* correlation was preferred over parametric Pearson's *r* to test the significance of relationships between independent variables.

Standard level of significance of *p* = 0.05 was adopted. Sequential Bonferroni correction was applied in cases of multiple comparisons. All statistical analyses were done using PAST Software, version 4.15 for MacOS [82].

## 3 Results

### 3.1 Survey Results

The survey included a total of 132 participants (Table S1), ranging from 41 having the boat moored in Gijón to 24 in Nalón marinas (Table S2). These respondents represented 6%, 15.5%, 13.5%, and 14.3% of the owners mooring in Gijón, Avilés, Nalón, and Ribadesella marinas, respectively.

In terms of fuel usage, 50.8% used petrol, 46.2% diesel, and 3.0% other or no fuel. For ship hull cleaning, 25.8% used ecological soap, 20.4% regular soap, and 53.8% other cleaning methods, generally pressurized water (Table S1). Water being not cytogenotoxic, we chose ecological soap (the second method in frequency) as an example of cleanser to test cytogenotoxicity experimentally.

The proportion of users of petrol (vs. the rest of fuels or not fuel) ranged from 38.7% in Avilés to 66.8% in Nalón marinas (Table S2). The difference between marinas was only marginally significant (*χ* ^2^ = 6.32, 3 df, *p* = 0.09; moderate Cramer's *V* = 0.22).

A greater proportion of boat owners used ecological soap in Avilés (35.5%), followed by Ribadesella (27.3%), Gijón (22.0%), and Nalón marinas (8.3%); while regular soap was employed by 12.2%, 6.5%, 33.3%, and 36.4%, respectively. Thus, most ship owners used pressurized water in all the marinas except Ribadesella. The difference between marinas for the boat cleaning method was significantly different (*χ* ^2^ = 17.55 with 6 df and *p* = 0.007; moderate Cramer's *V* = 0.24).

### 3.2 Induction of Nuclear Abnormalities by Experimental Exposure to Products Used in Marinas

The mean size of the limpets employed in the experiment was 2.75 mm (SD 0.29). Heavy metals were measured from water taken from the sampling site (El Rinconín beach). Cd, Hg, and Pb were not detected. Mean As concentration was 2.45 ppb (SD 1.15).

There was no mortality during the acclimatization period, except for one limpet from the tank further used to test the ecological soap. During the exposure time, samples from the petrol treatment exhibited the highest mortality rate (25%), followed by diesel (16.7%), ecological soap (9.1%), and the control group (0%).

The treatment that induced the highest MN frequency was petrol (mean 2.22‰ cells with MN), followed by diesel (0.90‰), ecological soap (0.80‰), and the control group (0.42‰) (Figure 3A). Kruskal–Wallis test was highly significant (*H* = 29.13, *p* < 0.001).

**FIGURE 3:**
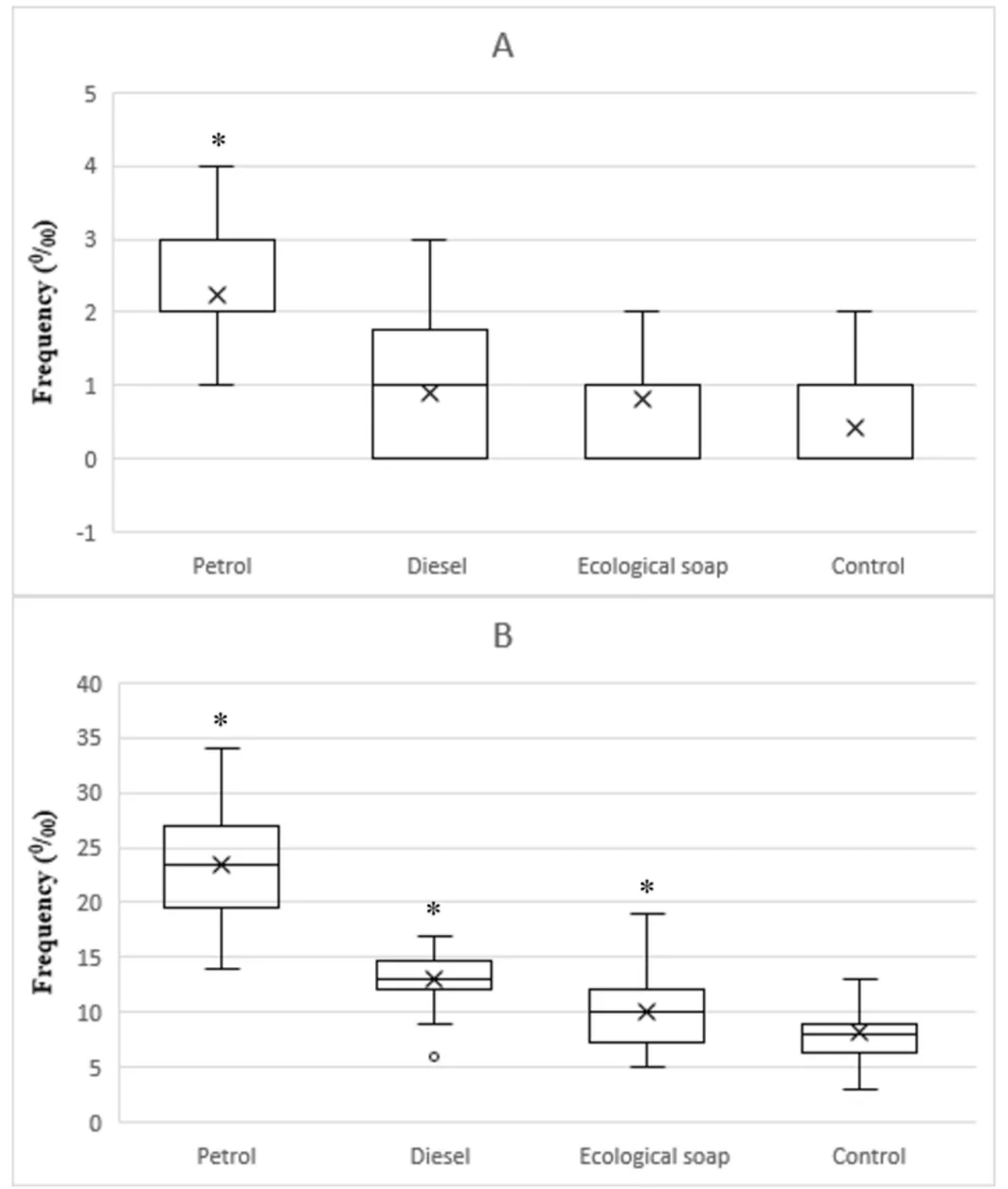
Boxplot of (A) MN and (B) the rest of nuclear abnormalities in gill cells per treatment. Results are given per 1000 cells. Asterisks indicate significant differences determined by the Mann–Whitney *U* test (*p* < 0.05).

The rest of nuclear abnormalities (Table S3) were more frequent than MN, oscillating between a mean of 8.08‰ in total in the control to 23.39‰ in the group treated with petrol (Figure 3B). Kruskal–Wallis test was highly significant (*H* = 54.24, *p* < 0.001).

Post hoc tests showed that the MN frequency in limpets treated with petrol was statistically different from the rest of treatments (Table 1A). However, the differences between the groups treated with diesel and ecological soap and the control were not significant (Table 1A). In contrast, the rest of the nuclear abnormalities differed significantly in all the pairs of comparisons except the group treated with ecological soap and the control (Table 1B), suggesting no significant genotoxicity of ecological soap at the concentration employed here.

**TABLE 1:** Pairwise Mann–Whitney test results (below diagonal) and their *p*‐values (above diagonal) for the frequency of (A) MN and (B) nuclear abnormalities in limpets exposed to different products.

	Petrol	Diesel	Ecosoap	Control
*(A)*				
Petrol		**1.59e−04**	**2.91e−05**	**3.65e−07**
Diesel	55		0.83	0.06
Ecosoap	42	192		0.08
Control	24	167	172	
*(B)*				
Petrol		**7.26e−07**	**4.11e−07**	**3.99e−08**
Diesel	10.5		**3.33e−03**	**1.91e−06**
Ecosoap	6.5	91.5		0.05
Control	0	38.5	157	

*Note:* Significant differences after Bonferroni correction are in bold. Ecosoap: ecological soap.

The proportion of the different abnormality types (Figure 4) did not differ between the control and the groups treated with diesel (*χ* ^2^ = 2.06, 4 df, *p* = 0.72; Cramer's *V* = 0.07) and ecological soap (*χ* ^2^ = 7.69, 4 df, *p* = 0.21; Cramer's *V* = 0.12). Only the group treated with petrol was significantly different from the control (*χ* ^2^ = 11.32 with 4 df and *p* = 0.023; Cramer's *V* = 0.14), exhibiting a lower proportion of binucleated cells (the most abundant type of the category “Other,” by far) and a higher proportion of MN and blebbed nuclei than the control and the rest of treated groups.

**FIGURE 4:**
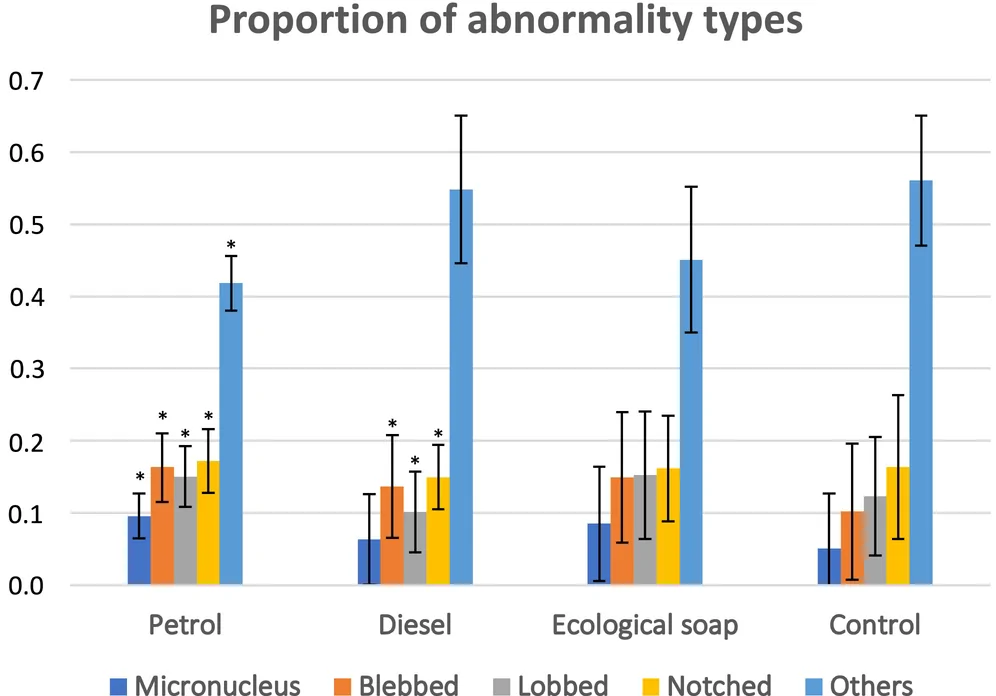
Proportion of the different types of nuclear abnormalities found in the four experimental groups. MN, micronuclei. The category “Other” contains principally binucleated cells. Asterisks indicate significant differences determined by the Mann–Whitney *U* test (*p* < 0.05).

### 3.3 Heavy Metal Pollution and Genotoxicity in Asturias Marinas

#### 3.3.1 Heavy Metal Content in Water

In water samples, heavy metal concentrations were similar in the sites examined (Table S4). As was the only trace element detected from all the sites analyzed. Cd, Hg, and Pb were detected only from Avilés marina, not surpassing the maximum concentrations allowed in the EU directive (0.2, 0.05, and 7.2 μg/L for Cd, Hg, and Pb, respectively; Directive 2008/105/EC). Note that the values in Table S4 are expressed in ppb. Therefore, comparisons between marinas could be done only with As.

Waters from Gijón marina exhibited the highest concentration of As, followed by Nalón, Avilés, and Ribadesella waters. ANOSIM test for As showed statistical differences between sites (*R* = 0.22, *p* = 0.048). Post hoc test showed differences between the least polluted Ribadesella and Gijón (*R* = 0.76, *p* = 0.03), also with Nalón (*R* = 0.38, *p* = 0.03). The average level of As in water was not significantly correlated with the number of moorings or the proportion of boats using petrol (*τ* = 0, *p* = 1 in both cases, n.s.), nor with the city population size (*τ* = 0.67, *p* = 0.17).

In El Rinconín beach, where the limpets for the experiment were taken from, the level of As was intermediate between the two most and the two least polluted sites (see results in Section 3.2).

#### 3.3.2 MN Test

The limpets with the highest MN frequency were found from Gijón marina (3.33‰), followed by Avilés (1.92‰), Nalón (1.38‰), and Ribadesella (1.17‰) (Figure 5A). The difference between ports was statistically significant; Kruskal–Wallis test with *H* = 27.25, *p* < 0.001. In post hoc tests, applying Bonferroni, the frequency of MN in limpets from Gijón was statistically significantly different from that of all the other sites (Table 2A). Ribadesella and Avilés were also significantly different, while Nalón, in the middle, did not differ from Avilés and Ribadesella (Table 2A).

**FIGURE 5:**
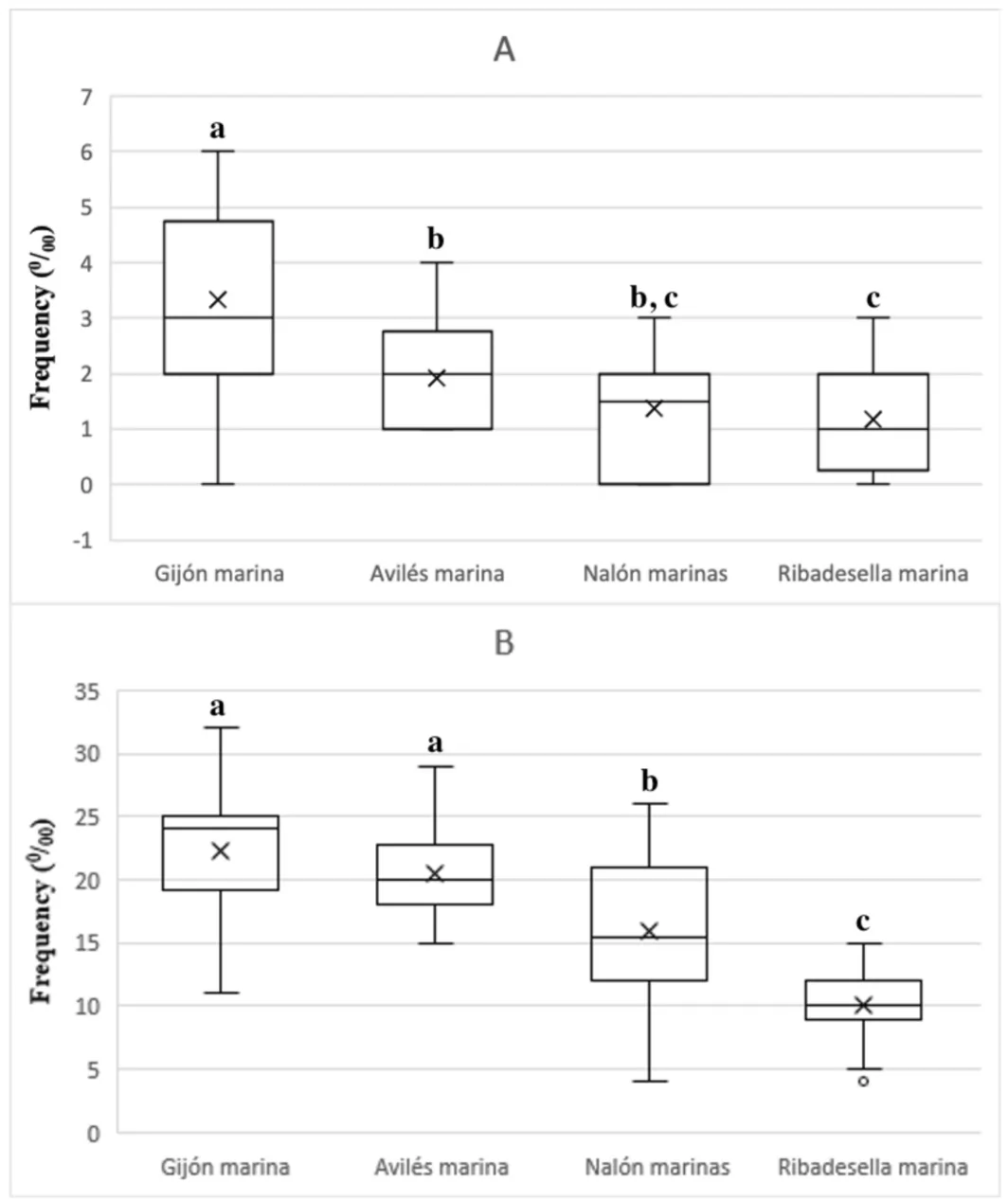
Boxplots of (A) micronuclei and (B) the sum of other nuclear abnormalities in limpet gill cells, per site. Results are given per 1000 cells. Groups with different letters are significantly different (Mann–Whitney *U* test, *p* < 0.05). Groups sharing the same letter do not differ significantly.

**TABLE 2:** Pairwise Mann–Whitney test results (below diagonal) and their *p*‐values (above diagonal) for the number of (A) MN and (B) sum of other nuclear abnormalities, in limpets from different marinas.

	Gijón	Avilés	Nalón	Ribadesella
*(A)*				
Gijón		**9.42e−04**	**5.05e−05**	**6.39e−06**
Avilés	131		0.13	**0.01**
Nalón	95	217		0.49
Ribadesella	72.5	171	255.5	
*(B)*				
Gijón		0.03	**4.60e−04**	**2.14e−08**
Avilés	185.5		**0.01**	**3.21e−09**
Nalón	118	165		**1.53e−04**
Ribadesella	16.5	1	104.5	

*Note:* Significant differences after Bonferroni correction are in bold.

Blebbed, lobed, and notched nuclei were more frequent than MN in all the marinas except in Gijón (Table S3), although the distributions of abnormality types in the limpets from the four marinas were not significantly different (*χ* ^2^ = 19.39, 12 df, *p* = 0.079; moderate Cramer's *V* = 0.063), with more anomalies of “Other” type in all the cases (principally binucleated cells as in the experiment).

The frequency of abnormalities other than MN (Figure 5B) was significantly different between the limpets sampled from the four marinas (*H* = 51.17, *p* < < 0.001). In post hoc pairwise tests, only limpets from Gijón and Avilés did not differ significantly from each other. The rest of the comparisons between marinas were significant (Table 2B).

We expected the products employed by the ships to be more abundant in the marinas with a higher number of ships using those products. From the experiment, the petrol induced a higher frequency of MN and nuclear abnormalities (Table 1), and a higher proportion of MN over the rest of abnormalities (Figure 4). Extrapolating the survey results to all the moored ships, the number of ships using petrol would be 77 in Avilés, 300 in Gijón, 137 in Nalón, and 147 in Ribadesella marinas. Following our expectation, limpets from the marinas with more ships using petrol exhibited a higher proportion of MN than those with fewer ships with petrol (Figure 6). The mean proportion of MN was 0.093 (SD 0.043) in Avilés, 0.148 (SD 0.058) in Gijón, 0.087 (SD 0.075) in Nalón, and 0.108 (SD 0.086) in Ribadesella. It was significantly different among marinas (Kruskal–Wallis *H* = 15.84 with *p* = 0.003). Applying Bonferroni correction Gijón and Ribadesella did not differ significantly from each other (Mann–Whitney *U* = 185, n.s.), nor Nalón and Avilés (Mann–Whitney = 271.5, n.s.). In contrast, MN proportion in Gijón limpets was significantly higher than those of Nalón and Avilés (*U* = 133.5 with *p* = 0.014 and *U* = 126 with *p* = 0.009, respectively). Ribadesella, intermediate between Gijón and the other two marinas, did not differ significantly from them (*U* = 257.5 with Avilés and *U* = 249 with Nalón, both n.s.). Moreover, the relationship between the recreational boats and the proportion of MN was supported by a significant correlation between the latter variable and the number of moorings in the marinas (*τ* = 1, *p* = 0.041).

**FIGURE 6:**
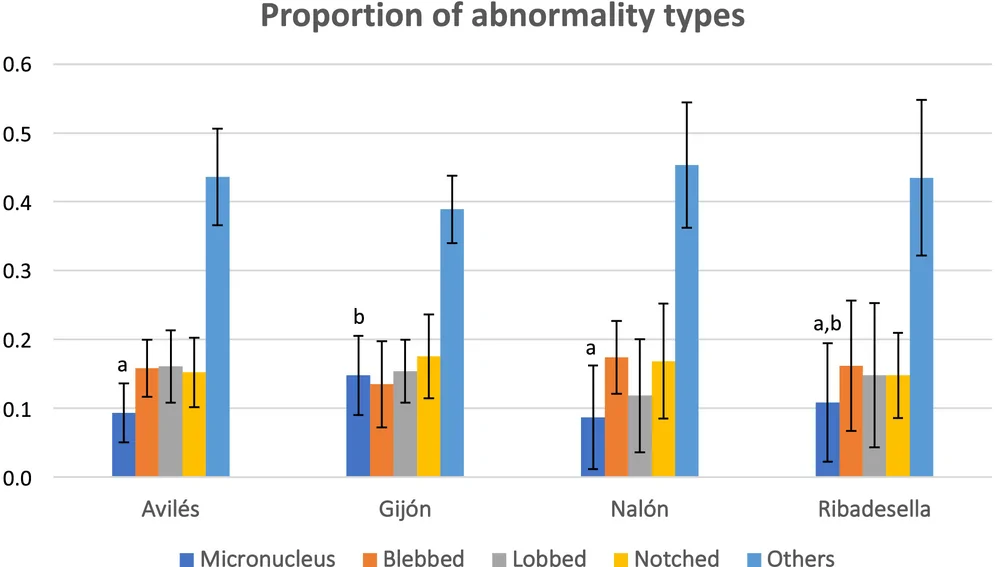
Proportion of the different types of nuclear abnormalities in limpets sampled from the marinas analyzed, taking all the individuals together. MN: micronuclei. The category “Others” contains principally binucleated cells. Groups with different letters are significantly different (Mann–Whitney *U* test, *p* < 0.05). Groups sharing the same letter do not differ significantly.

Regarding the associations between biological and environmental variables, in the LSR analysis the population size significantly explained the mean MN counts, while the mean of other nuclear abnormalities was explained from the disturbance score (Table 3), driven by the proportion of notched nuclei and secondarily the lobed ones that were the abnormalities significantly explained by this score. Significant regressions with other environmental variables were not found, only a marginally significant regression of MN and maritime traffic (Table 3). The rest of the regressions, all not significant, are not shown. The proportion of boats using diesel was also tested and gave results far from significance.

**TABLE 3:** Ordinary least square regressions to test relationships between different types of nuclear abnormalities and environmental variables.

Variables		Regression				
Dependent	Independent	Slope a (SE)	Intercept *b* (SE)	*r* ^2^	*t*	*p*
Mean MN	Population size	126 230 (8669)	−15 670 (18 608)	0.991	14.561	0.0047
	Moorings	229.99 (63.41)	−125.01 (134.64)	0.868	3.627	0.068
Other abnormalities (sum)	Disturbance score	0.233 (0.037)	3.499 (0.662)	0.952	6.266	0.024
Notched	Disturbance score	1.156 (0.198)	4.224 (0.593)	0.944	5.826	0.028
Lobed	Disturbance score	1.373 (0.317)	3.982 (0.848)	0.904	4.336	0.049

*Note:* Only significant or marginally significant associations are presented.

## 4 Discussion

The results obtained in this study revealed significant differences between nuclear abnormalities in limpets from marinas with different levels of maritime traffic estimated from the number of moorings, according to the departure hypothesis. The proportion of MN was higher in limpets from the marinas with more boats using petrol; this suggests a plausible relationship between recreational boating and cytogenotoxicity. However, in the regression analysis, other factors were significant, with maritime traffic explaining only marginally MN count means. The human population size explained MN better; this could be expected, since higher cytogenotoxicity has been associated with urban concentration in zones as diverse as Brazil [81] or Madagascar [80]. Moreover, the sum of disturbance scores explained other nuclear abnormalities, notched and lobed nuclei, that are related to aneugenic toxicity [47, 48]. From our results, recreational shipping is a factor contributing to genotoxicity, and this is a novelty of this study. However, the size of the neighboring population was also a significant variable in our study. This illustrates a complex situation with different factors at play producing genotoxicity in marinas.

In the results from the exposure experiment, petrol stood out as the most genotoxic product among those assayed in this study (Figure 3) and caused the highest mortality rate (25%). Petrol has been pointed out as a dangerous genotoxic contaminant of marine environments [83, 84, 85], primarily for containing trace metals as Pb [85]. Moreover, other studies pointed out the additive effect in the genotoxicity of Pb when combined with other elements such as As [86], a metal abundant in Asturias waters [87]. Diesel also exhibited some genotoxic activity, although to a lesser extent than petrol. This result agrees with other studies [16]. Genotoxic activity from diesel primarily arises from the PAHs produced during its combustion [88]. Still, this genotoxic activity is lower compared with heavy metals, although there is an additive effect when mixed with Pb [86]. In the diesel used in this study, there was no information on heavy metals content, but since Pb in diesel is expected to be lower‐to‐none [20] we can assume the genotoxicity was not due to this metal. Since no combustion was performed during the experiment, the main genotoxic activity of diesel could not be assessed [89]. This was not the case for petrol, as Pb genotoxic activity occurs whether it has undergone a combustion process or not [24]. Regarding the group treated with ecological soap, in our experiment, there was no difference in MN and other nuclear abnormalities when compared with the control group. This is consistent with previous studies where ecological soaps exhibited lower genotoxic activity than conventional commercialized soaps [90]. Ecological soap could be a safe cleaning product to use in marinas.

In the results of in situ sampling, as commented above there is likely a mixture of factors inducing cytogenotoxicity. The differences in cytogenetic abnormalities between marinas were not entirely consistent with the number of moorings or even the concentration of heavy metals detected in water. Limpets from the Gijón marina exhibited the highest frequency of MN and nuclear abnormalities. This result could be associated with a higher boat traffic in the marina, with more moorings than the rest, and probably also to a dense maritime traffic in the surroundings because the neighboring Gijón port is the largest and most active port in the Asturias region [65]. At the same time, Gijón was also the marina most polluted with As, and this element is a well‐known cause of increased MN frequency [91]. It is difficult to disclose the individual effects of As pollution and spills from boats with the present data.

Limpets from the Avilés marina were next, exhibiting a similar frequency of nuclear abnormalities as Gijón but significantly higher than those from the Nalón and Ribadesella marinas, despite sheltering even fewer moorings than Ribadesella. Likely, the other toxic heavy metals found in Avilés water (Cd and Hg, in addition to Pb) contribute to increased nuclear abnormalities. Sources of toxic pollutants could be the adjacent fishing port and the international commercial port, and the significant industrial activity in Avilés through the 20th century [92]. Cases of dark placentas were reported from Avilés between 1970 and 1980 [93], likely caused by the large amount of black carbon particles emitted by the industries [94]. All this pollution affected the ecosystem [95], enriching the nearby sediments in Cd, Hg, and Pb [96]. Thus, Avilés results could be again explained from a combination of pollution sources.

Far from Gijón and Avilés regarding the cytogenotoxicity indicators here reported, limpets from the Nalón marinas were third in the rank of MN and other nuclear abnormalities, exhibiting a higher frequency of nuclear abnormalities than Ribadesella limpets although more boats use petrol in the latter marina. In this case, a high concentration of As could be the reason. A possible explanation for that concentration could be the historical mining in the Nalón basin, whose impacts in terms of heavy metal pollution can still be observed today [97]. Finally, limpets from the Ribadesella marina (the second in number of moorings and boats using petrol) exhibited the fewest frequency of MN and nuclear abnormalities, and the lowest As content in water. Possibly there are fewer pollution sources there, at least of heavy metals, because the activities in the basin are mainly agriculture and tourism, with far less industry and mining than in the other sites. Although Cd pollution derived from agriculture practices had been reported in sediments and fish from River Sella [98], traces of Cd were not found in the marina waters studied.

It is important to note that this study did not distinguish the effects of heavy metals from those of PAHs; thus, future research should include controlled laboratory exposures to isolate the specific impacts of each. In any case, the cellular abnormalities observed in limpet tissues indicate a significant genotoxic and cytotoxic impact associated with fuel pollution, particularly in regions near larger human populations. Petrol is posing risks to this ecologically and economically important mollusk. These results emphasize the necessity for monitoring and conservation of this keystone species of intertidal habitats [58] and sustainably managing it as a local resource.

Promoting the use of diesel engines over petrol engines on boats, or, much better, the use of renewable energy through financial incentives or awareness‐raising campaigns would be advised, as more than 50% of survey respondents use petrol engines (69 out of 133 respondents). Public awareness campaigns are strongly recommended, as they have proven to be effective in the past for environmental conservation efforts [99]. These campaigns could lead to more informed decision‐making among boat owners and operators. Ultimately, these efforts can contribute to more sustainable practices in the ports.

The levels of genetic damage in marine fauna associated with the ports of the Bay of Biscay deserve further investigation, potentially employing additional genetic damage biomarkers and assessing physical, chemical, and microbiological variables tied to urban and industrial effluent from densely populated areas, where MN rate is higher as indicated in our study. A panel of biomarkers, such as the comet assay, gene expression analysis, and the MN test, could be applied for evaluating the effects of chemical pollution on marine organisms in the Bay of Biscay, so enabling for an early detection of health risks within these populations.

A limitation of this study was the lack of temporality. The change in pollution input levels can affect the MN test [100]; perhaps in summer, in the middle of the touristic season, cytogenotoxicity could be higher in Nalón and Ribadesella marinas for more wastes but also for more movements of the boats. Other authors pointed out the existence of a seasonality pattern in MN frequencies in mussels [101]; it could happen in limpets as well. To our knowledge, there is no previous literature about seasonality or the effect of exposure time on MN levels in limpets. Future research could tackle these issues with seasonal samplings and different exposure periods. Also, our experiment lasted only for 5 days. It is possible that more cytogenotoxic activity could appear if the exposure time were longer, although it does not seem very likely [102]; in any case, given the high mortality found in our experiment, a longer duration would encompass a serious decrease in the sample size, reducing the statistical power. Finally, there are few data about MN in limpets [61]. Future studies should perform the MN assays in different limpets to corroborate and better contextualize the results from this study.

## 5 Conclusions

The results of this study confirm that recreational boating, and, in particular, the size of the population, contributes to cytogenotoxicity in limpets, mainly through fuel spills and associated pollutants.

Petrol used in boats exhibited a higher level of cytogenotoxic activity compared to diesel, whereas ecological soap showed no cytogenotoxic activity. This suggests that ecological soap may be a viable, less harmful alternative for boat cleaning, showing no significant genotoxic impact.

Limpets from the Gijón marina exhibited the highest frequency of MN and nuclear abnormalities, followed by those from the Avilés, Nalón, and Ribadesella marinas, respectively. Launching public awareness campaigns as a mitigation strategy to enhance the usage of diesel over petrol in boats is recommended.

Further research is necessary to explore the effects of seasonality on MN levels in limpets. This will help to ensure that our results accurately reflect the true impact of genotoxicity on limpet populations in the Asturias marinas.

## Author Contributions

**Carlos Valiente‐Diaz:** conceptualization, data curation, formal analysis, investigation, methodology, visualization, writing – original draft, writing – review and editing. **Eva Garcia‐Vazquez:** conceptualization, funding acquisition, project administration, resources, supervision, validation, visualization, writing – review and editing. **Verónica Soto‐López:** conceptualization, project administration, resources, supervision, validation, writing – review and editing. All authors have read and agreed to the published version of the manuscript.

## Funding

This research was supported by the Ministry of Science, Innovation and Universities of Spain, Grant PID2022‐138523OB‐100, and the Government of Asturias Principality, Grant GRU‐GIC‐24‐051. We thank the staff of the marinas and the many boat owners that kindly answered our questions in the survey.

## Conflicts of Interest

The authors declare no conflicts of interest.

## Supporting information


**Table S1:** Summary of surveys' answers. N.A.: not answered.
**Table S2:** Summary of the survey results. The number of respondents and the proportion of them that use each type of product are given. The population inhabiting in each site and the number of fixed moorings (summing the two marinas in the case of Nalón) are also provided.
**Table S3:** Mean and standard deviation (in brackets) of nuclear abnormalities in gill cells. Results are expressed per 1000 cells.
**Table S4:** Description of limpet samples and mean heavy metal concentration in water (in ppb; SD in brackets). *N*: number of samples collected. ND: not detected. Length is expressed in cm (standard deviation in brackets).

## Data Availability

The data that support the findings of this study are available from the corresponding author upon reasonable request.
