# Supplementary material for: Micronuclei and Other Nuclear Abnormalities in Patella Limpet as Biomarkers of Cytogenotoxicity in Recreational Port Waters
Source: Environ Toxicol. 2026 Feb 23;41(11):1125–38. doi: 10.1002/tox.70055 (PMC13656886; doi:10.1002/tox.70055)
Supplement: Supplementary file 1 [file TOX-41-1125-s001.docx]

**SUPPLEMENTARY MATERIAL**

**Supplementary Table 1**. Summary of surveys’ answers. N.A.: Not answered.

| **Site** | **Boat type** | **Fuel type** | **Boat hull cleaning products** | **Cleaner** |
| --- | --- | --- | --- | --- |
| Site 1 | Sailboat | Petrol | Acid Multicleaner | The owner |
| Site 1 | Sailboat | Petrol | Nothing in particular | The owner |
| Site 1 | Sailboat | N.A. | N.A. | The owner |
| Site 1 | Sailboat | N.A. | Soap | The owner |
| Site 1 | Recreational boat with less than 8 mts length | Diesel | Products used in the shipyards | Shipyard |
| Site 1 | Sailboat |  | Soap | The owner |
| Site 1 | Sailboat | Petrol | Products used in the shipyards | Shipyard |
| Site 1 | Sailboat | Petrol | Pressurized water | The owner |
| Site 1 | Sailboat | Diesel | Nothing in particular | The owner |
| Site 1 | Sailboat | Diesel | Acid Multicleaner | The owner |
| Site 1 | Sailboat | Diesel | Soap | The owner |
| Site 2 | Sailboat | Petrol | Products used in the shipyards | Shipyard |
| Site 2 | Recreational boat with less than 8 mts length | Petrol | Products used in the shipyards | Shipyard |
| Site 2 | Sailboat | Petrol | Products used in the shipyards | Shipyard |
| Site 2 | Sailboat | Petrol | Acid Multicleaner and Soap | The owner |
| Site 2 | Sailboat | Diesel | Pressurized water | The owner |
| Site 2 | Recreational boat with less than 8 mts length | Diesel | Products used in the shipyards | Shipyard |
| Site 2 | Sailboat | Diesel | Pressurized water | The owner |
| Site 2 | Recreational boat with less than 8 mts length | Diesel | Products used in the shipyards | Shipyard |
| Site 2 | Sailboat | Diesel | Products used in the shipyards | Shipyard |
| Site 2 | Sailboat | Diesel | Products used in the shipyards | Shipyard |
| Site 2 | Recreational boat with less than 8 mts length | Diesel | Ecological soap | The owner |
| Site 4 | Sailboat | Petrol | Acid Multicleaner | The owner |
| Site 2 | Sailboat | Diesel | Products used in the shipyards | Shipyard |
| Site 2 | Sailboat | Diesel | Products used in the shipyards | Shipyard |
| Site 2 | Sailboat | Diesel | Pressurized water | The owner |
| Site 2 | Sailboat | Diesel | Products used in the shipyards | Shipyard |
| Site 2 | Sailboat | Diesel | Products used in the shipyards | Shipyard |
| Site 1 | Sailboat | Diesel | Pressurized water | The owner |
| Site 2 | Recreational boat with more than 8 mts length | Diesel | Products used in the shipyards | Shipyard |
| Site 1 | Sailboat | Diesel | Soap | The owner |
| Site 1 | Sailboat | Diesel | Pressurized water | The owner |
| Site 1 | Recreational boat with less than 8 mts length | Petrol | Pressurized water | The owner |
| Site 1 | Sailboat | Petrol | In the shipyard and Acid Multicleaner | Shipyard |
| Site 1 | Sailboat | Diesel | Products used in the shipyards | Shipyard |
| Site 1 | Recreational boat with less than 8 mts length | Petrol | Ecological soap | The owner |
| Site 1 | Recreational boat with less than 8 mts length | Petrol | Acid Multicleaner and Ecological soap | The owner |
| Site 1 | Recreational boat with less than 8 mts length | Petrol | Products used in the shipyards | Shipyard |
| Site 1 | Sailboat | Diesel | Products used in the shipyards | Shipyard |
| Site 3 | Recreational boat with less than 8 mts length | Petrol | Soap and Pressurized water | The owner |
| Site 3 | Sailboat | Diesel | Acid Multicleaner and Ecological soap | The owner |
| Site 3 | Recreational boat with less than 8 mts length | Petrol | Pressurized water | The owner |
| Site 3 | Recreational boat with less than 8 mts length | Petrol | Soap | The owner |
| Site 3 | Sailboat | Petrol | N.A. | The owner |
| Site 3 | Recreational boat with less than 8 mts length | Petrol | Pressurized water | The owner |
| Site 3 | Recreational boat with less than 8 mts length | Petrol | Patent | The owner |
| Site 2 | Sailboat | Diesel | Pressurized water and Sanding | The owner |
| Site 3 | Sailboat | Diesel | Pressurized water | Shipyard |
| Site 3 | Sailboat | Diesel | Pressurized water | The owner |
| Site 3 | Sailboat | Diesel | Pressurized water | Shipyard |
| Site 3 | Recreational boat with less than 8 mts length | Petrol | Pressurized water | The owner |
| Site 4 | Recreational boat with less than 8 mts length | Petrol | Ecological soap and pressurized water | The owner |
| Site 2 | Sailboat | Petrol | Patent | The owner |
| Site 1 | Sailboat | Petrol | Pressurized water | The owner |
| Site 4 | Recreational boat with less than 8 mts length | Petrol | Soap and Pressurized water | The owner |
| Site 4 | Recreational boat with less than 8 mts length | Petrol | Ecological soap and Pressurized water | The owner |
| Site 1 | Sailboat | Diesel | Water and sanding | The owner |
| Site 4 | Recreational boat with less than 8 mts length | Petrol | Soap and water | The owner |
| Site 4 | Recreational boat with more than 8 mts length | Petrol | Soap and water | The owner |
| Site 4 | Sailboat | Diesel | Ecological soap | The owner |
| Site 4 | Sailboat | Diesel | Cleaning products used in the shipyard | Shipyard |
| Site 4 | Recreational boat with more than 8 mts length | Petrol | Soap and Pressurized water | The owner |
| Site 4 | Recreational boat with less than 8 mts length | Petrol | Ecological soap and Acid Multicleaner | The owner |
| Site 4 | Recreational boat with less than 8 mts length | Petrol | Pressurized water | The owner |
| Site 4 | Sailboat | Diesel | Acid Multicleaner | The owner |
| Site 4 | Sailboat | Diesel | Pressurized water | The owner |
| Site 4 | Recreational boat with less than 8 mts length | Petrol | Pressurized water | The owner |
| Site 4 | Sailboat | Diesel | Soap and Pressurized water | The owner |
| Site 4 | Sailboat | Diesel | Soap and Pressurized water | The owner |
| Site 4 | Recreational boat with less than 8 mts length | Diesel | Cleaning products used in the shipyard | Shipyard |
| Site 4 | Recreational boat with more than 8 mts length | Petrol | Soap and Pressurized water | The owner |
| Site 4 | Recreational boat with more than 8 mts length | Diesel | Soap and Water | The owner |
| Site 4 | Recreational boat with less than 8 mts length | Petrol | Pressurized water | The owner |
| Site 4 | Recreational boat with less than 8 mts length | Petrol | Soap and Pressurized water | The owner |
| Site 3 | Recreational boat with less than 8 mts length | Diesel | Manual | The owner |
| Site 3 | Sailboat | Diesel | Soap and Pressurized water | The owner |
| Site 3 | Recreational boat with less than 8 mts length | Petrol | Ecological soap | The owner |
| Site 3 | Recreational boat with less than 8 mts length | Petrol | Soap and Pressurized water | The owner |
| Site 3 | Sailboat | Diesel | Soap and Pressurized water | The owner |
| Site 3 | Recreational boat with less than 8 mts length | Petrol | Soap and Water | The owner |
| Site 2 | Recreational boat with less than 8 mts length | Diesel | Products used in the shipyards | Shipyard |
| Site 2 | Sailboat | Diesel | Ecological soap | The owner |
| Site 2 | Recreational boat with less than 8 mts length | Petrol | Ecological soap | The owner |
| Site 2 | Sailboat | Diesel | Ecological soap | The owner |
| Site 2 | Sailboat | Diesel | Ecological soap | The owner |
| Site 2 | Sailboat |  | Ecological soap | The owner |
| Site 2 | Recreational boat with less than 8 mts length | Petrol | Soap and Pressurized water | The owner |
| Site 2 | Recreational boat with less than 8 mts length | Petrol | Ecological soap | The owner |
| Site 2 | Recreational boat with less than 8 mts length | Petrol | Ecological soap | The owner |
| Site 2 | Recreational boat with less than 8 mts length | Petrol | Ecological soap | The owner |
| Site 2 | Recreational boat with less than 8 mts length | Petrol | Ecological soap | The owner |
| Site 2 | Recreational boat with less than 8 mts length | Petrol | Ecological soap and Acid Multicleaner | The owner |
| Site 3 | Recreational boat with less than 8 mts length | Petrol | Pressurized water | Shipyard |
| Site 3 | Recreational boat with less than 8 mts length | Petrol | Soap and Pressurized water | The owner |
| Site 4 | Recreational boat with less than 8 mts length | Petrol | Soap and Pressurized water | The owner |
| Site 4 | Recreational boat with less than 8 mts length | Petrol | Soap and Pressurized water | The owner |
| Site 4 | Sailboat | Diesel | Ecological soap and Pressurized water | The owner |
| Site 4 | Recreational boat with less than 8 mts length | Petrol | Pressurized water | The owner |
| Site 4 | Recreational boat with less than 8 mts length | Diesel | Pressurized water | The owner |
| Site 4 | Recreational boat with less than 8 mts length | Diesel | Ecological soap and Pressurized water | The owner |
| Site 3 | Recreational boat with less than 8 mts length | Petrol | Pressurized water | The owner |
| Site 3 | Recreational boat with less than 8 mts length | Petrol | Soap and Pressurized water | The owner |
| Site 3 | Sailboat | Diesel | Pressurized water | The owner |
| Site 3 | Recreational boat with less than 8 mts length | Petrol | Pressurized water | The owner |
| Site 3 | Recreational boat with less than 8 mts length | Petrol | Pressurized water | The owner |
| Site 1 | Sailboat | Diesel | Cleaning products used in the shipyard | Shipyard |
| N.A. | Sailboat | Diesel | Ecological soap, Pressurized water and Soap | The owner |
| N.A. | Sailboat | Diesel | Cleaning products used in the shipyard | Shipyard |
| Site 1 | Recreational boat with less than 8 mts length | Petrol | Cleaning products used in the shipyard | Shipyard |
| Site 1 | Sailboat | Diesel | Cleaning products used in the shipyard | Shipyard |
| Site 4 | Recreational boat with less than 8 mts length | Petrol | Pressurized water | The owner |
| Site 4 | Recreational boat with less than 8 mts length | Petrol | Ecological soap and Pressurized water | The owner |
| Site 4 | Recreational boat with less than 8 mts length | Petrol | Soap and Pressurized water | The owner |
| Site 4 | Sailboat | Diesel | Pressurized water | The owner |
| Site 4 | Recreational boat with less than 8 mts length | Petrol | Ecological soap and Pressurized water | The owner |
| Site 4 | Recreational boat with less than 8 mts length | Petrol | Ecological soap | The owner |
| Site 1 | Sailboat | Diesel | Acid Multicleaner and Pressurized water | The owner |
| Site 1 | Sailboat | Diesel | Cleaning products used in the shipyard | Shipyard |
| Site 1 | Sailboat | Diesel | Ecological soap and Pressurized water | The owner |
| Site 1 | Recreational boat with less than 8 mts length | Petrol | Soap and Pressurized water | The owner |
| Site 1 | Sailboat | Diesel | Ecological soap | The owner |
| Site 1 | Sailboat | Diesel | Acid Multicleaner | The owner |
| Site 1 | Recreational boat with less than 8 mts length | Petrol | Cleaning products used in the shipyard | Shipyard |
| Site 1 | Sailboat | Diesel | Pressurized water and Sanding | The owner |
| Site 1 | Recreational boat with less than 8 mts length | Petrol | Ecological soap and water | The owner |
| Site 1 | Recreational boat with less than 8 mts length | Petrol | Cleaning products used in the shipyard | Shipyard |
| Site 1 | Sailboat | Diesel | Ecological soap | The owner |
| Site 1 | Sailboat | Diesel | Ecological soap | The owner |
| Site 1 | Recreational boat with less than 8 mts length | Petrol | Cleaning products used in the shipyard | Shipyard |
| Site 1 | Sailboat | Petrol | Ecological soap and Pressurized water | The owner |
| Site 1 | Sailboat | Petrol | Ecological soap and Acid Multicleaner | The owner |
| Site 1 | Sailboat | Diesel | Cleaning products used in the shipyard | Shipyard |

**Supplementary Table 2.** Summary of the survey results. The number of respondents and the proportion of them that use each type of product are given. The population inhabiting in each site and the number of fixed moorings (summing the two marinas in the case of Nalón) are also provided.

| **Site** | **City population** | **Fixed moorings** | **Answers** | **Petrol as fuel (%)** | **Diel as fuel (%)** | **Ecological soap**  **(%)** | **Regular soap (%)** |
| --- | --- | --- | --- | --- | --- | --- | --- |
| Gijón | 268,313 | 683 | 41 | 43.90 | 48.78 | 21.95 | 12.20 |
| Avilés | 75,518 | 200 | 31 | 38.71 | 58.06 | 35.48 | 6.45 |
| Nalón | 7,235 | 178 | 24 | 66.67 | 33.33 | 8.33 | 33.33 |
| Ribadesella | 5,548 | 231 | 33 | 63.63 | 36.36 | 27.27 | 36.36 |

**Supplementary Table 3**. Mean and standard deviation (in brackets) of nuclear abnormalities in gill cells. Results are expressed per 1000 cells.

| **Group** | **Micronucleus** | **Blebbed** | **Lobed** | **Notched** | **Other** | **All** |
| --- | --- | --- | --- | --- | --- | --- |
| **Marinas** |  |  |  |  |  |  |
| Gijón | 3.33  (1.61) | 2.88 (1.30) | 3.33 (1.09) | 4.04 (1.83) | 8.63 (2.16) | 22.21 (5.06) |
| Avilés | 1.92  (0.97) | 3.21 (0.88) | 3.29 (1.20) | 3.08 (1.06) | 8.92 (1.95) | 20.42 (3.12) |
| Nalón | 1.38  (1.10) | 2.75 (1.36) | 2.08 (1.50) | 2.79 (1.61) | 6.96 (2.37) | 15.96 (5.98) |
| Ribadesella | 1.17  (0.96) | 1.58 (0.78) | 1.54 (1.02) | 1.42 (0.65) | 4.38 (1.31) | 10.08 (2.78) |
| **Experiment** |  |  |  |  |  |  |
| Petrol | 2.22  (0.81) | 3.72 (1.02) | 3.56 (1.34) | 4.11 (1.45) | 9.78 (2.21) | 23.39 (5.14) |
| Diesel | 0.90  (0.91) | 1.80 (1.01) | 1.35 (0.75) | 1.95 (0.69) | 7.05 (1.50) | 13.05 (2.56) |
| Ecological soap | 0.80  (0.77) | 1.55 (1.00) | 1.60 (1.10) | 1.70 (0.86) | 4.45 (1.57) | 10.10 (3.60) |
| Control | 0.42  (0.65) | 0.88 (0.85) | 1.04 (0.75) | 1.29 (0.81) | 4.46 (1.35) | 8.08 (2.45) |

**Supplementary Table 4**. Description of limpet samples and mean heavy metal concentration in water (in ppb; SD in brackets). N: number of samples collected. N.D.: not detected. Length is expressed in cm (standard deviation in brackets).

| **Site** | **Limpets** | | **Trace elements in water samples** | | | |
| --- | --- | --- | --- | --- | --- | --- |
|  | N | Mean length | As | Cd | Hg | Pb |
| #1 Gijón marina | 12 | 3.31 (0.37) | 2.88 (0.87) | N.D. | N.D. | N.D. |
| #2 Avilés marina | 12 | 2.77 (0.18) | 1.76 (0.88) | 0.31 (0.20) | 0.15 (0.11) | 2.97 (2.51) |
| #3 Nalón marinas | 12 | 3.21 (0.25) | 2.58 (1.32) | N.D. | N.D. | N.D. |
| #4 Ribadesella marina | 12 | 2.98 (0.28) | 1.01 (0.29) | N.D. | N.D. | N.D. |
